# Emerging role of Jumonji domain-containing protein D3 in inflammatory diseases

**DOI:** 10.1016/j.jpha.2024.100978

**Published:** 2024-04-16

**Authors:** Xiang Li, Ru-Yi Chen, Jin-Jin Shi, Chang-Yun Li, Yan-Jun Liu, Chang Gao, Ming-Rong Gao, Shun Zhang, Jian-Fei Lu, Jia-Feng Cao, Guan-Jun Yang, Jiong Chen

**Affiliations:** a State Key Laboratory for Managing Biotic and Chemical Threats to the Quality and Safety of Agro-products, Ningbo University, Ningbo, Zhejiang, 315211, China; b Laboratory of Biochemistry and Molecular Biology, School of Marine Sciences, Ningbo University, Ningbo, Zhejiang, 315211, China; c Ningbo No. 2 Hospital, Ningbo, Zhejiang, 315211, China; d China Ningbo Institute of Life and Health Industry, University of Chinese Academy of Sciences, Ningbo, Zhejiang, 315211, China

**Keywords:** JMJD3, Inflammation, Histone demethylation, Inhibitor

## Abstract

Jumonji domain-containing protein D3 (JMJD3) is a 2-oxoglutarate-dependent dioxygenase that specifically removes transcriptional repression marks di- and tri-methylated groups from lysine 27 on histone 3 (H3K27me2/3). The erasure of these marks leads to the activation of some associated genes, thereby influencing various biological processes, such as development, differentiation, and immune response. However, comprehensive descriptions regarding the relationship between JMJD3 and inflammation are lacking. Here, we provide a comprehensive overview of JMJD3, including its structure, functions, and involvement in inflammatory pathways. In addition, we summarize the evidence supporting JMJD3's role in several inflammatory diseases, as well as the potential therapeutic applications of JMJD3 inhibitors. Additionally, we also discuss the challenges and opportunities associated with investigating the functions of JMJD3 and developing targeted inhibitors and propose feasible solutions to provide valuable insights into the functional exploration and discovery of potential drugs targeting JMJD3 for inflammatory diseases.

## Highlights

• JMJD3 is an H3K27me2/3 demethylase mediating transcriptional activation of associated genes.
• JMJD3 is implicated in various physiological and pathological processes, including inflammation.
• JMJD3 is a potential therapeutic target for multiple inflammatory diseases.
• No JMJD3 inhibitor is clinically available up to now.

## Graphical abstract

**Figure:**
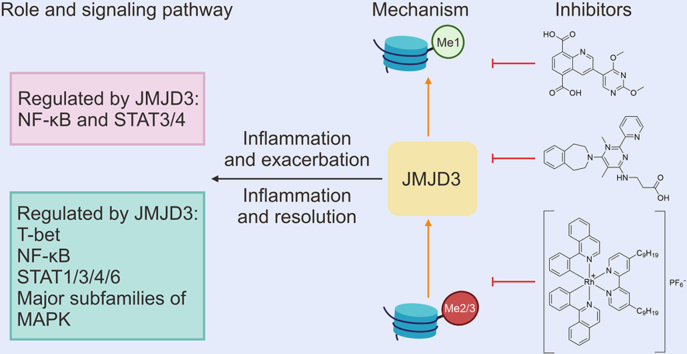

## 1 Introduction

Epigenetics pertains to heritable alterations in gene function that occur without altering the DNA sequences [1,2]. These modifications are susceptible to environmental hazard factors, lifestyle, and nutritional status [3]. Histone methylation, a vital epigenetic alteration, is implicated in mediating chromatin remodeling and thereby modulating the expression of associated genes [4]. Histone methylation is also involved in many innate and adaptive immune responses [5].

Trimethylated lysine 27 on histone 3 (H3K27me3) is a key epigenetic modification associated with gene repression, whereas H3K27me1 typically implies activated gene expression [6]. Jumonji domain-containing protein D3 (JMJD3) belongs to the lysine-specific demethylase 6 (KDM6) subfamily that specifically removes di- and trimethylated groups from histone H3K27 to activate the expression of targeted genes [7,8]. Inflammation is a protective response of the host to infections and tissue damage, aiding in preventing pathogen transmission, and promoting pathogen clearance and wound healing [9]. Inflammation is beneficial to the host when it resolves over time, whereas inflammatory dysregulation leads to various inflammatory diseases [10]. JMJD3 extensively plays crucial roles in immune diseases, infectious diseases, tumorigenesis, apoptosis, and cellular differentiation by targeting specific transcription factors and epigenetic regulators [11]. JMJD3 is generally maintained at a low level in tissues under homeostatic conditions, whereas it is strongly induced by various cellular stress stimuli [[12], [13], [14]]. Various inflammatory mediators and stress inducers can trigger JMJD3 expression, leading to the modulation of inflammatory diseases through diverse signaling pathways [6]. However, systematic studies that comprehensively describe the relationship between inflammation and JMJD3 expression are lacking. This summary highlights JMJD3's critical involvement in the initiation and progression of inflammation, explores the signaling pathways associated with JMJD3, and discusses the potential use of JMJD3 inhibitors in treating different inflammatory conditions. These insights shed light on JMJD3's roles in inflammatory disease pathogenesis and its potential as a therapeutic target against inflammation-related disorders.

## 2 Structure and function of JMJD3

**Fig. 1:**
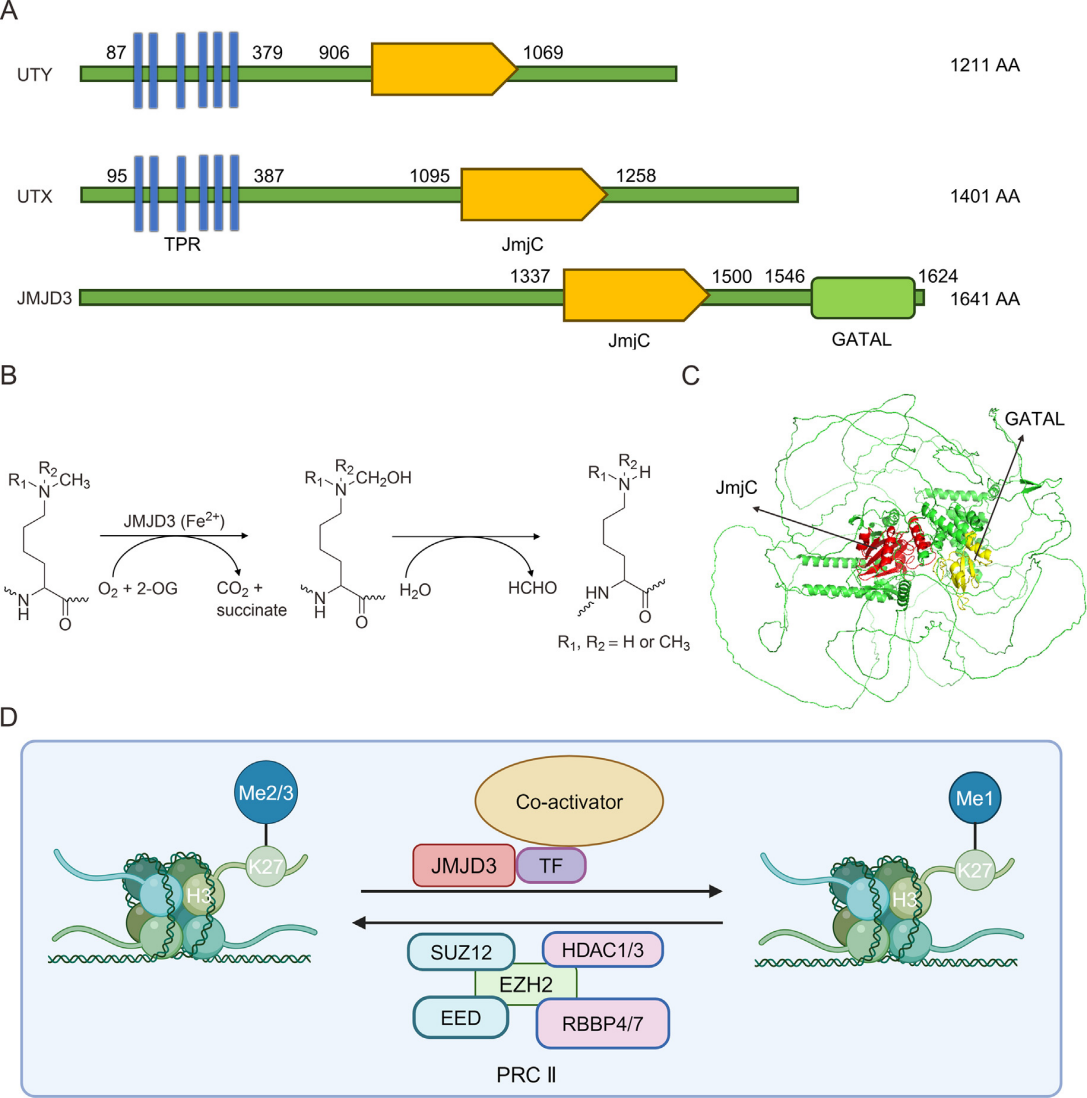
The structure and catalytic mechanism of Jumonji domain-containing protein D3 (JMJD3). (A) Key information on three members of the lysine-specific demethylase 6 (KDM6) family. (B) The catalytic mechanism of JMJD3. (C) The structure of JMJD3 is predicated by AlphaFold, and its Jumonji C (JmjC) and GATA-like (GATAL) domains are labeled with red and yellow colors, respectively. (D) A visual depiction of the mechanisms by which JMJD3 regulates and boosts gene transcription. UTY: ubiquitously transcribed tetratricopeptide repeat (TPR), Y chromosome; AA: amino acid; UTX: ubiquitously transcribed TPR, X chromosome; TPR: translocated promoter region; 2-OG: 2-oxo-glutarate; TF: transcription factors; SUZ12: suppressor of zest 12; HDAC1/3: histone deacetylase 1/3; EZH2: enhancer of zeste homolog 2; EED: embryonic ectoderm development; RBBP4/7: retinoblastoma binding protein 4; PRC II: polycomb repressive complex 2.

JMJD3 is a 2-oxo-glutarate (2-OG)- and Fe^2+^-dependent demethylase belonging to the KDM6 subfamily, which includes three members: JMJD3; ubiquitously transcribed tetratricopeptide repeat (TPR), X chromosome (UTX); and ubiquitously transcribed TPR, Y chromosome (UTY). Notably, the mammalian UTX and UTY genes reside on the X and Y chromosomes, respectively [15]. Structurally, all three, UTY, JMJD3, and UTX, have Jumonji C (JmjC) structural domains, but UTY does not have the ability to catalyze H3K27-specific demethylase activity compared to JMJD3 and UTX. Interestingly, JMJD3 can only catalyze H3K27me2/3 compared to UTX which can catalyze H3K27me1/2/3, which may be due to the lack of TPR structural domain in JMJD3 and a dearth of this domain in UTX abrogating its activity for mono-demethylation [16] (Figs. 1A−C). The human *JMJD3* gene is located on chromosome 17p13.1 and encodes a polypeptide containing 1,682 amino acid residues [17]. Functionally, JMJD3 adds O_2_ to the substrate-methylated amino groups on lysine residues of histones with the participation of cofactors Fe^2+^ and 2-OG to generate one molecule of succinate, one molecule of CO_2_, and a hydroxylated intermediate, which subsequently produces unstable semialdehyde that further decomposes into demethylated lysine and formaldehyde [15] (Figs. 1B and D). Apart from the JmjC domain, there is a Zn^2+^-coordinated GATA-like (GATAL) domain situated within a four-helix bundle formed by α-helical segments in the C-terminal region of JMJD3, and this domain is indispensable for demethylase activity and C-terminal stability of the truncated JMJD3 protein [18] (Fig. 1C). Moreover, JMJD3 exhibits demethylase-independent activity by recruiting and interacting with different coactivators to occupy *cis*-acting elements of downstream genes and activate their transcription [5]. Furthermore, JMJD3 was found to induce the degradation of plant homeodomain finger protein 20 (PHF20) via recruiting Trim26 without relying on its H3K27 demethylase function [19]. Given the presence of numerous post-translational modification (PTM) sites on other KDMs, which are known to regulate their activities both related and unrelated to demethylation [4,8,20,21], it is reasonable to speculate that PTMs on JMJD3 itself are crucial in controlling its varied roles (Table 1).

**Table 1:** Post-translational (PTM) modifications of Jumonji domain-containing protein D3 (JMJD3). The PTM sites are predicated by The Cuckoo Workgroup (http://biocuckoo.cn/index.php).

Phosphorylation	SUMOylation	Methylation	Palmitoylation	Propionylation	Lysine acetylation	Ubiquitination
Y1631	K991	R192	C1138	K991	K228	K1004
Y425	K168	R369	C990	K168	K1004	K1008
Y320	K228	R586	C921	K228	K1033	K1011
Y75	K1180	V1152	C817	K1180	K168	K769
Y1493	K1013	R1272	C96	K1013	K191	K977
Y313	K1008	R1551	C972	K1008	K1008	K1169
Y1189	K1011	R1595	C1605	K1011	K185	K1175
Y576	K981	K89	C1547	K981	K1013	K1180
T1310	K1296	R198	C1602	K1296	K1279	K1321
S621	K487	K191	C491	K487	K1011	K805

SUMOylation: small ubiquitin-like modifier mediated modification.

## 3 JMJD3-mediated inflammatory pathways

**Fig. 2:**
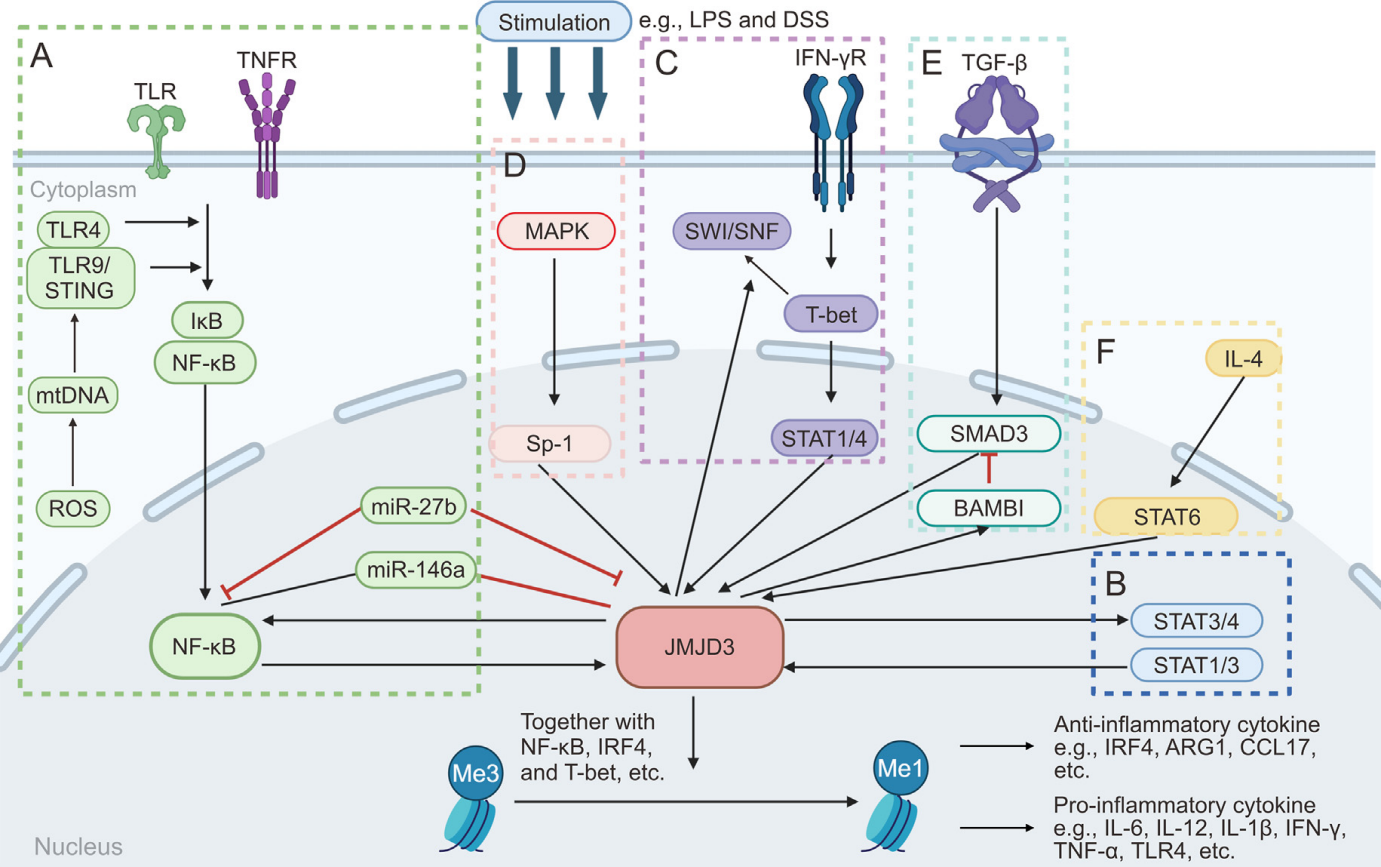
A diagram introducing the regulatory network associated with Jumonji domain-containing protein D3 (JMJD3). JMJD3 can be upregulated by different signaling pathways, including (A) nuclear factor-kappa B (NF-κB) signaling, (B) signal transducer and activator of transcription (STAT) signaling, (C) T-bet signaling, (D) major subfamilies of mitogen-activated protein kinase (MAPK) signaling, (E) transforming growth factor-β (TGF-β)/SMAD family member 3 (SMAD3) signaling, and (F) interleukin-4 (IL-4)/STAT6/interferon (IFN) regulatory factor 4 (IRF4) signaling. Then JMJD3 is recruited to the chromatin via interacting with several transcription factors. JMJD3 activates the transcription of pro- and anti-inflammatory genes by demethylating the repressive trimethylated lysine 27 on histone 3 (H3K27me3) markers on their promoters and gene bodies. ROS: reactive oxygen species; mtDNA: mitochondrial DNA; TLR: Toll-like receptor; TNFR: tumor necrosis factor (TNF) receptor; STING: stimulator of IFN genes; miR-27b: microRNA-27b; LPS: lipopolysaccharides; DSS: dextran sulfate sodium; Sp-1: specificity protein 1; IFN-γR: IFN-gamma receptor; SWI/SNF: switch/sucrose nonfermentable; BAMBI: bone morphogenetic protein (BMP) and activin membrane bound inhibitor; ARG1: arginase 1; CCL17: C–C motif chemokine ligand 17.

The JMJD3-mediated inflammatory pathways play crucial roles in regulating the expression of genes that promote inflammation, as well as in directing polarization of immune cells (Fig. 2). Targeting these pathways may lead to the development of novel anti-inflammatory interventions for treating inflammatory disorders.

### 3.1 Nuclear factor-kappa B (NF-κB) signaling

NF-κB signaling is involved in various key stages of inflammation via regulating numerous cellular processes involved in inflammation progression, such as the production of inflammatory factors, differentiation of effector and regulatory T (Treg) cells, and maturation of dendritic cells, etc. [22,23]. The promoter region of *JMJD3* consists of two conserved κB sites, which suggests it is associated with NF-κB signaling [12]. Notably, JMJD3 also stimulates the expression of NF-κB, which forms a positive feedback loop that promotes its transcription [24] (Fig. 2A). Thus, JMJD3 is implicated in NF-κB-mediated signaling. In lipopolysaccharide (LPS)-treated bone marrow-derived macrophages (Mфs), JMJD3 is recruited to the promoters of thousands of genes, but only inducible inflammatory genes are suppressed when JMJD3 is knocked down [12,25]. In LPS-treated human umbilical vein endothelial cells (HUVECs), JMJD3-NF-κB signaling was activated and thus raised the levels of pro-inflammatory genes tumor necrosis factor-α (*TNF-α*), matrix metalloprotease-9 (*MMP-9)*, interleukin-1β (*IL-1β*), *IL-6*, and cyclooxygenase-2 (*COX-2*), and finally contributed to vascular inflammation as in atherosclerosis (AS) [26]. In an interesting observation from studies on peritoneal Mфs isolated from mice with sepsis, JMJD3 and NF-κB exhibited opposing effects on the regulation of anti-inflammatory microRNA-146a (*miR-146a*). Specifically, while the suppression of NF-κB leads to a decrease in miR-146a levels, inhibiting JMJD3 resulted in an increased expression of this microRNA (miRNA) [27]. This finding highlights the complex regulatory roles that JMJD3 and NF-κB play in the immune response, particularly in the modulation of anti-inflammatory mechanisms within the context of sepsis. Notably, miR-27b, another miRNA delivered by exosomes derived from mesenchymal stem cells (MSCs), suppressed the levels of both JMJD3 and NF-κB and thus alleviated sepsis by reducing inflammatory responses. This finding suggests that the delivery of miR-27b via MSC-derived exosomes has therapeutic potential against sepsis [28]. The involvement of the JMJD3-NF-κB pathway has been highlighted by the increased presence of JMJD3 in osteoarthritis (OA)-affected cartilage. Targeting JMJD3 for inhibition has shown significant outcomes, notably reducing the levels of genes associated with inflammation and catabolism, as well as diminishing the degradation of collagen II and aggrecan during IL-1β-induced stress in laboratory settings. Moreover, inhibiting JMJD3 has also been effective in protecting against cartilage damage caused by DMM in animal models. These discoveries point to the potential of JMJD3 inhibition as a strategic method for alleviating cartilage deterioration in OA, suggesting a promising avenue for therapeutic intervention in this condition [29]. JMJD3 is a pivotal regulator of Mф polarization and inflammation in the pathogenesis of abdominal aortic aneurysms (AAA) in both human and murine models. Its mechanism of action involves the removal of H3K27me3 from NF-κB inflammatory gene promoters in Mфs, thereby contributing to adverse vascular remodeling, aortic dilation, and AAA progression. Consequently, targeting JMJD3 represents a potential therapeutic strategy for mitigating pathological Mф-driven inflammation, thereby attenuating AAA progression and reducing the risk of rupture. Nevertheless, further elucidation of the precise molecular mechanisms involved and rigorous translational studies are warranted to ascertain the clinical implications and therapeutic viability of JMJD3 modulation in AAA management [30]. Chronic unpredictable mild stress (CUMS) and maternal separation induce depression-like behaviors in animal models. These stressors also lead to microglial activation via activating JMJD3-NF-κB signaling and raising corresponding pro-inflammatory cytokines in the prefrontal cortex (PFC) and hippocampus (HIP) [[31], [32], [33]]. In acute pancreatitis (AP), JMJD3 is upregulated in the pancreas and lungs, potentially because of the presence of mitochondrial DNA (mtDNA) or oxidized mtDNA as a result of tissue injury caused by AP. The release of mtDNA and oxidized-mtDNA contributes to the infiltration of inflammatory monocytes in lung injury through the activation of the stimulator of IFN genes (STING) and Toll-like receptor 9 (TLR9) signaling pathways, ultimately leading to the activation of NF-κB and subsequent upregulation of JMJD3 and TNF-α. However, inhibiting JMJD3 or using *Jmjd3*-knockout (KO) mice has been shown to alleviate pulmonary inflammation induced by AP. Additionally, blocking mtDNA oxidation or silencing the TLR9/STING pathway effectively reduces inflammatory responses. These findings suggest that targeting JMJD3 or inhibiting the STING/TLR9 pathway may hold therapeutic potential for the treatment of AP and associated lung injuries [34]. When bacterial infection occurs, it leads to the release of LPS, which activates the TLR4-NF-κB pathway, resulting in the induction of JMJD3 expression. The increased levels of JMJD3 subsequently upregulate the expressions of CCAAT/enhancer-binding protein β (C/EBPβ) and KDM5A. Notably, KDM5A exerts its regulatory influence by repressing the expression of IL-4-induced gene-1 (IL-4i1) through demethylation of H3K4me3, thereby promoting polarization towards M1-like/M2-like phenotypes and exacerbating inflammatory responses [35]. In summary, the JMJD3 and NF-κB pathways exert a notable influence on the regulation of inflammation. Delving deeper into this pathway could present the potential for the development of innovative therapeutic approaches for inflammatory conditions. However, comprehensive research is required to fully understand these intricacies and their potential clinical implications.

### 3.2 Signal transducer and activator of transcription (STAT) 1 signaling

JMJD3 is involved in the STAT1, STAT3, and STAT4 signaling pathways (Fig. 2B), which play pivotal roles in immune responses and inflammation [36,37]. The *JMJD3* promoter contains multiple p-STAT1 and p-STAT3 binding sites, indicating that STAT1 and STAT3 regulate JMJD3 expression by occupying its promoter. Additionally, STAT1 and STAT3 interact with the promoters of certain JMJD3-mediated inflammatory genes such as IL-6, C–C motif chemokine ligand 5 (*CCL5*), and interferon (IFN) regulatory factor 7 (*IRF7*), thereby augmenting their expression [36]. JMJD3 regulates the expression of genes involved in T helper (Th) 1 cell differentiation by modulating the binding of STAT3 and STAT4 to H3K27me3. Specifically, JMJD3 is involved in controlling the transcription of IL-12 induced by STAT3 and STAT4 [37]. Furthermore, JMJD3 has been reported to serve as a link mediating the crosstalk between STAT and TLR signalings via NF-κB signaling in microglia. Upon activation of TLR4, NF-κB signaling is initiated, leading to the phosphorylation and subsequent transcriptional activity of STAT1 and STAT3 in tissues. Both NF-κB and STAT signaling pathways contribute to the upregulation of JMJD3 expression, further promoting the upregulation of pro-inflammatory mediators [38]. Reportedly, glycoprotein nonmetastatic melanoma protein B (GPNMB) may impact IL-6 expression and apoptosis in human periodontal ligament cells (hPDLCs) by influencing JMJD3 through the TLR4-IκB and STAT3 pathways [39]. Furthermore, INF-β regulates JMJD3 expression via the Janus kinase (JAK)/STAT signaling pathway, subsequently inducing NF-κB-mediated inflammatory gene transcription in infiltrating aortic Mфs [30]. JMJD3 also regulates inflammation in Mфs recruited to wounds by reducing H3K27me3 on inflammatory gene promoters, such as STING, through the JAK1/JAK3/STAT3 pathway. Furthermore, JMJD3 was observed to upregulate STAT3, ionized calcium-binding adaptor molecule-1, and IL-1β protein levels via vestigial-like family member 4 (VGLL4) in LPS-treated microglia [40]. Furthermore, JMJD3 was found to upregulate STAT3 and IL-17A expression in dextran sulfate sodium (DSS)-induced colonic tissue [41] and promote the occurrence of inflammation in the colon through the NF-κB and JAK2/STAT3 pathways [42]. Overall, the comprehensive roles of JMJD3 in modulating immune responses and inflammation through its interactions with the STAT1, STAT3, and STAT4 signaling pathways highlight the intricate regulatory mechanisms that govern these critical biological processes. Further research is essential to gain a deeper understanding of the precise molecular pathways and functional significance of JMJD3 in immune regulation and inflammatory responses.

### 3.3 T-bet signaling

T-bet is a key transcription factor belonging to the T-box family of transcription factors and is implicated in the differentiation and maintenance of Th1 cells, a subset of CD4^+^ T cells mediating cellular immune responses [43]. Th1 cells are involved in immune defense by secreting the cytokine IFN-γ, which in turn activates phagocytosis and reactive oxygen species (ROS) production in Mфs to combat pathogen invasion [5]. JMJD3 plays a significant role in the differentiation and migration of T cells via facilitating the interaction between the T-bet and Brahma-related gene 1, a component of the switching/sucrose non-fermenting (SWI/SNF) chromatin remodeling complex [44]. The complex disrupts the histone-DNA interaction through its ATPase activity, leading to activating Th1 target genes, including the *IFN-γ* gene. Notably, JMJD3-induced activation of T-bet target genes occurred independently of its demethylase activity [45]. Subsequently, T-bet recruits STAT4 and histone methyltransferases to facilitate H3K4me2 methylation. This epigenetic alteration augments the expression of genes related to Th1 cell differentiation and function [46]. Furthermore, apart from its function in promoting Th1 differentiation, T-bet suppresses the differentiation of Th2 cells by impeding the activity of Th2 master regulators such as GATA binding protein 3 (GATA3) and Bcl6 through JMJD3. Specifically, deletion of JMJD3 inhibits Th1 cell differentiation [5,47]. In summary, JMJD3 and T-bet pathways play significant roles in the modulation of inflammation, particularly in the context of Th1 and Th2 cell differentiation (Fig. 2C). Delving deeper into this pathway could yield valuable insights for developing therapeutic strategies targeting inflammatory diseases.

### 3.4 Major subfamilies of mitogen-activated protein kinase (MAPK) signaling

The MAPK, namely p38, extracellular signal-regulated kinase (ERK), and Jun N-terminal kinase (JNK) subfamilies, are activated by pathogen infection/tissue damage via stimulating pattern recognition receptors located on the cell surface and within the cytoplasm of immune cells [48]. Despite the fact that MAPK signaling encompasses almost most aspects of the inflammatory network, there are relatively limited investigations on the relationship between JMJD3 and MAPK [6]. JMJD3 regulates synoviocyte-mediated fibroblast-like proliferation and joint destruction in rheumatoid arthritis (RA). JMJD3 is activated by ERK/MAPK signaling, leading to increased expression of the proliferating cell nuclear antigen (*PCNA*) gene through demethylase activity, ultimately resulting in an aggressive RA phenotype [49]. IL-1β upregulates JMJD3 through the involvement of the specificity protein 1 (Sp-1) and MAPK signalings. The increased JMJD3 levels lead to the reduction of H3K27me3's occupations on the *TLR2* promoter, subsequently resulting in the upregulation of pro-inflammatory genes related to RA and arthritis [50]. Overall, JMJD3 and MAPK pathways are potential therapeutic targets for inflammatory diseases (Fig. 2D). However, more studies are imperative to fully explore the potential of targeting JMJD3 and MAPK signalings in this context, as studies on this topic are currently limited.

### 3.5 Transforming growth factor-β (TGF-β)/SMAD family member 3 (SMAD3) signaling

TGF-β, a cytokine primarily produced by M2-like Mфs, is known for its anti-inflammatory properties [51,52]. However, it also elicited pro-inflammatory effects through interactions with JMJD3 (Fig. 2E). The TGF-β signaling pathway significantly influences inflammation by modulating Th cell differentiation and reducing JMJD3-mediated production of cytokines such as IL-2, IL-4, and IFN-γ, as evidenced in systemic sclerosis (SSc) [53]. Additionally, TGF-β can activate multiple signaling pathways, including the SMAD3 pathway. Mounting evidence indicates that JMJD3 is implicated in regulating the SMAD3-mediated TGF-β signaling pathway [54,55]. During neurogenesis in neural stem cells, JMJD3 modulates the expression of a cluster of genes responsive to TGF-β via influencing the interaction between JMJD3 and SMAD3 in the promoter regions of these genes [56]. In SSc skin and experimental fibrosis, JMJD3 levels are increased in fibroblasts in a TGF-β/SMAD3 signaling-dependent manner. The upregulation of JMJD3 leads to fibroblast activation by inducing the expression of Fos-related antigen 2 (FRA2) [57]. Furthermore, in diabetic nephropathy (DN) renal mesangial cells, elevated levels of TGF-β have been found to upregulate pro-fibrotic and inflammatory genes such as connective tissue growth factor (*Ctgf)*, *Serpine1*, and *CCL2*. This upregulation is mediated by TGF-β-induced expression of JMJD3 and miR-101b, which inhibits the expression of enhancer of zeste homolog 2 (EZH2) [58]. In hepatic stellate cells, a study revealed that EZH2 and JMJD3 play a role in regulating HSC activation, partially through epigenetic modulation of the expression of bone morphogenetic proteins and activin membrane-bound inhibitors (BAMBI). BAMBI serves as a crucial negative regulator of TGF-β signaling and can form a ternary complex with SMAD7 and the TGF-β type I receptor, thereby impeding SMAD3 activation and inhibiting signal transduction [59]. Moreover, JMJD3 and pro-inflammatory cytokines IL-17A, IL-21, and IL-22 were found to be upregulated, while TGF-β and IL-10 were downregulated in a DSS-induced mouse colitis model. This observation suggests that JMJD3 may exert a negative regulatory effect on TGF-β expression in this particular context [41].

### 3.6 IL-4/STAT6/IRF4 signaling

Mфs possess the ability to polarize into distinct phenotypes in response to environmental stress. The two primary phenotypes observed are classically activated (M1-like) and alternatively activated (M2-like) Mфs. M1-like Mфs can be activated by several factors, such as TLR ligands, granulocyte-Mф colony-stimulating factor (GM-CSF), and Th1-type cytokines. This activation results in the release of several cytokines such as TNF-α, IL-1β, IL-6, and IL-23. In contrast, the activation of M2-like Mфs is triggered by various factors including Mф colony-stimulating factor (M-CSF), immune complexes, Th2-type cytokines IL-4 and IL-13, and complement components. M2-like Mфs secrete numerous anti-inflammatory proteins such as IL-4, IL-10, and TGF-β, which dampen immune responses and promote tissue repair in multiple diseases. Polarization of Mфs into M1-like or M2-like phenotypes is a dynamic process that allows them to adapt their functions in response to specific environmental cues and contributes to immune regulation and tissue homeostasis [[60], [61], [62]]. JMJD3 plays a significant role in guiding the M2-like Mф response during helminth infection *in vivo*. It promotes the expression of IRF4, a potent transcriptional inducer of the alternative M2-like phenotype, in a demethylase-dependent manner [63]. Furthermore, treatment with IL-4, a cytokine known to induce M2-like Mф polarization, upregulates the levels of both JMJD3 protein and M2-like marker genes in Mфs derived from mouse bone marrow. IL-4-dependent expression of JMJD3 is mediated by STAT6 [64]. A separate study demonstrated that the upregulation of CCL17 expression during IL-4-induced M2-like Mф formation was mediated through the JMJD3-IRF4 pathway. Inhibition of STAT6 not only suppresses CCL17 expression induced by IL-4 but also hinders the production of JMJD3 and IRF4 in human monocytes [65]. IL-4 treatment could induce M2-like polarization of liver Mфs (namely Kupffer cells) in donor livers via activating the STAT6-JMJD3 pathway and suppressing the TLR-4-NF-κB pathway. However, another study has indicated that the monocytes/Mфs *in vitro* are activated by the GM-CSF/JMJD3/IRF4/CCL17 pathway. These findings suggested that CCL17 may have additional functions beyond its known chemotactic role [66,67] (Fig. 2F). The activation of the circuit-α7 nicotinic acetylcholine receptor (α7nAChR), which is encoded by the *Chrna7* gene, has been found to enhance the IL-4 induced expression of arginase 1 (ARG1) in Mфs. This process involves the activation of STAT6 and the JMJD3-H3K27me3 methylation pathways [68]. IFN-β effectively inhibits the JMJD3-IRF4-dependent pathway in Mфs activated by GM-CSF and IL-4. This inhibition occurs through the regulation of the 2-OG/succinate ratio [69]. In addition, 2-OG is essential for the activation and polarization of M2-like Mфs. When M2-like Mфs are activated, 2-OG is engaged in fatty acid oxidation and the JMJD3-dependent epigenetic reprogramming of M2-like genes. Conversely, during polarization of M2-like Mфs, 2-OG accumulation leads to JMJD3-dependent demethylation at H3K27me3 [70,71].

## 4 Role of JMJD3 in inflammation

**Fig. 3:**
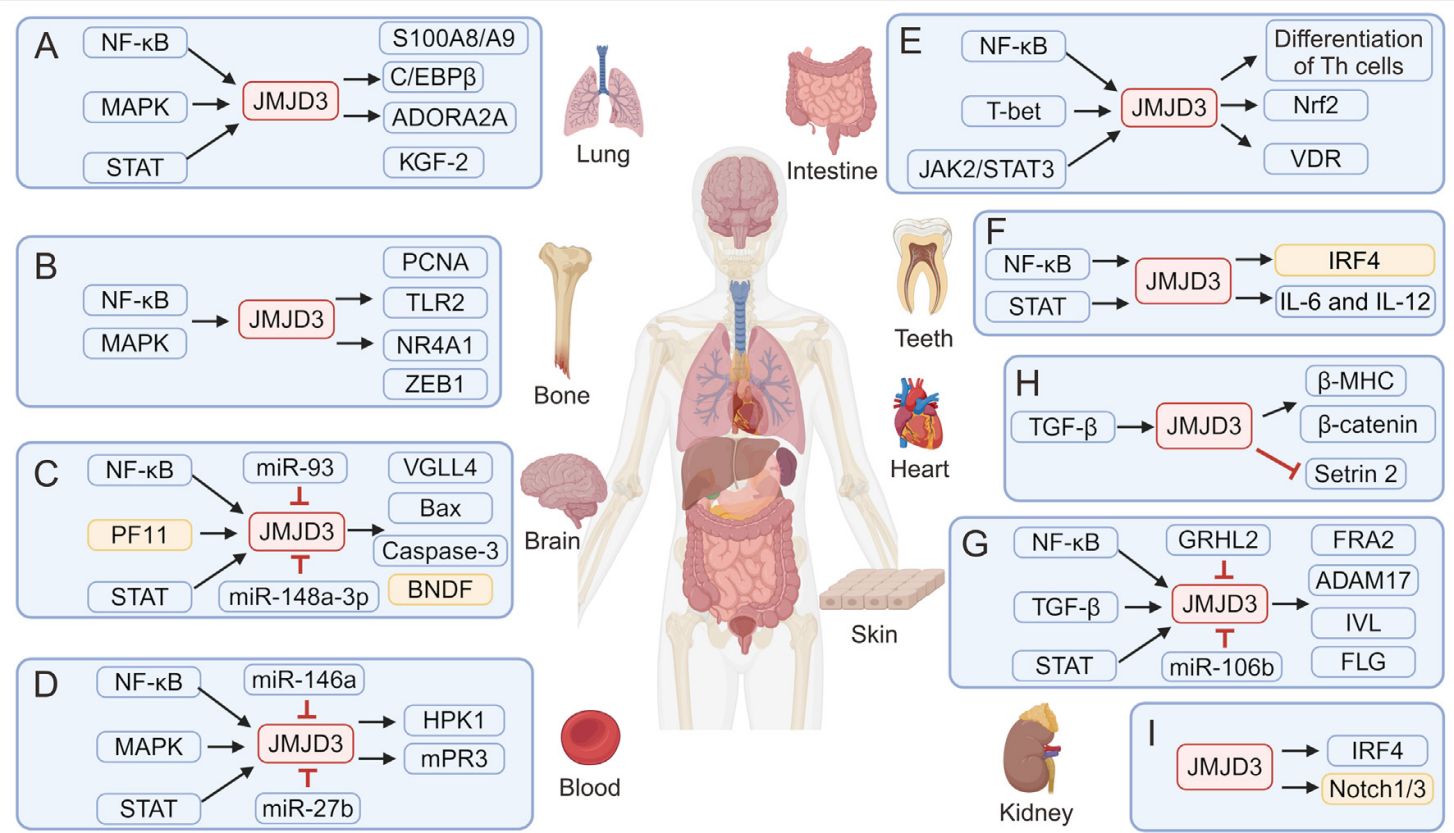
The role of Jumonji domain-containing protein D3 (JMJD3) in inflammation. JMJD3 is a histone demethylase enzyme that can be upregulated by various inflammatory cytokines. It exerts its influence on gene expression, thereby influencing pro- or anti-inflammatory responses in diverse organs including the (A) lung, (B) bone, (C) brain, (D) blood, (E) intestine, (F) teeth, (G) skin, (H) heart, and (I) kidney. NF-κB: nuclear factor-kappa B; MAPK: major subfamilies of mitogen-activated protein kinase; STAT: signal transducer and activator of transcription; S100A8/A9: S100 calcium binding protein A8/A9; C/EBPβ: CCAAT/enhancer-binding protein β; ADORA2A: adenosine aA2aA receptor; KGF-2: keratinocyte growth factor-2; PCNA: proliferating cell nuclear antigen; TLR2: Toll-like receptor 2; NR4A1: nuclear receptor subfamily 4 group A member 1; ZEB1: E-box binding homeobox 1; PF11: pseudoginsenoside-F11; miR-93: microRNA-93; VGLL4: vestigial-like family member 4; Bax: B-cell lymphoma 2 (BCL2) associated X; BNDF :brain-derived neurotrophic factor; HPK1: hematopoietic progenitor kinase 1; mPR3: membrane proteinase 3; JAK2: Janus kinase 2; Th: T helper; Nrf2: nuclear factor erythroid-2-related factor-2; VDR: vitamin D receptor; IRF4: interferon regulatory factor 4; IL: interleukin; TGF-β: transforming growth factor-β; β-MHC: beta-myosin heavy chain; GRHL2: grainyhead-like 2; FRA2: Fos-related antigen 2; ADAM17: a disintegrin and metalloproteinase 17; IVL: involucrin; FLG: filaggrin.

JMJD3 is implicated in modulating inflammatory responses, particularly in Mф polarization and immune responses. Its dysregulation is believed to be involved in various inflammatory disorders including RA, inflammatory bowel disease, and AS (Table 2 [27,28,30,34,35,40,47,49,50,57,61,68,[72], [109]] and Fig. 3). Therefore, targeting JMJD3 is a potential therapeutic strategy for modulating inflammatory responses and treating inflammatory disorders.

**Table 2:** The functions of Jumonji domain-containing protein D3 (JMJD3) in inflammatory diseases.

Organs	Diseases	Regulator of JMJD3	Regulated by JMJD3	Refs.
Lung	ALI	NF-κB^+^, MAPK^+^, and STAT^+^	S100A8/A9^+^, ADORA2A^+^, C/EBPβ^+^, Nrf2^−^, and IL-4il^−^	[35,72,73]
	Others	STING^+^, TLR9^+^, and NF-κB^+^	TNF-α^+^, ARG1^+^, and KGF-2^−^	[34,68,74]
Bone	RA	PDGF^+^, IL-1β^+^, MAPK^+^, and Sp-1^+^	PCNA^+^ and TLR2^+^	[49,50]
	OA	NF-κB^+^, IL-1β^+^, and HMGA1^+^	NR4A1^+^ and ZEB1^+^	[75,76]
Brain	Depression	NF-κB^+^ and STAT3^+^	HMGA1^+^ and VGLL4^+^	[40]
	CSI	miR-93^−^, miR-148a-3p^−^, ATF4^+^, and PF11^+^	Bax^+^, Caspase-3^+^, KLF2^+^, JUNB^+^, and ETS1^+^	[61,[77], [78], [79]]
	AD	–	BDNF^+^	[80,81]
Spinal cord	SCI	NF-κB^+^	MMP-3^+^ and MMP-9^+^	[82,83]
Blood	Sepsis	MAPK^+^ and STAT^+^	mPR3^+^, IL-1β^+^, miR-27b^+^, miR-146a^+^, and CSE/H_2_S^+^	[27,28,84,85]
	AS	NF-κB^+^ and IFN-β^+^	–	[30]
	SLE	NF-κB^+^, USP7^+^, and miR-1246^−^	HPK1^+^, CD11a^+^, and CREMα^+^	[[86], [87], [88], [89]]
Intestine	Colitis	NF-κB^+^	Foxp3^+^, CD44^+^, CXCR3^+^, Nrf2^+^, RORc^−^, GATA3^−^, and RALDH^−^	[47,[90], [91], [92]]
Teeth	PD	NF-κB^+^ and GPNMB^+^	IL-6^+^, IL-12^+^, STAT3^+^, and IRF4^+^	[[93], [94], [95], [96]]
Skin	SSc	–	FRA2^+^, ADAM17^+^, and Jagged-1^+^	[57,97]
	Hyperproliferative skin diseases	–	IVL^+^, KRT1^+^, FLG^+^, LCE^+^, and GRHL2^−^	[98]
	Diabetic wounds	NF-κB^+^, TLR4^+^, IL-6^+^, STAT3^+^, and miR-106b^−^	TNF-α^+^, IL-1β^+^, Notch1^+^, RhoU^+^, and PLAU^+^	[[99], [100], [101]]
Muscle	Heart disease	NF-κB^+^	β-MHC^+^, Sestrin2^+^, and β-catenin^+^	[[102], [103], [104]]
	Chronic cystitis	–	CCND1^+^ and COL1/3^+^	[105]
Kidney	CKD	IL-4^+^	IRF4^+^ and Notch1/3^+^	[[106], [107], [108], [109]]

ALI: acute lung injury; NF-κB: nuclear factor-kappa B; MAPK: major subfamilies of mitogen-activated protein kinase; STAT: signal transducer and activator of transcription; S100A8/A9: S100 calcium binding protein A8/A9; ADORA2A: adenosine aA2aA receptor; C/EBPβ: CCAAT/enhancer-binding protein β; Nrf2: nuclear factor erythroid-2-related factor-2; IL-4il: interleukin-4-induced gene-1; STING: stimulator of interferon (IFN) genes; TLR9: Toll-like receptor 9; TNF-α: tumor necrosis factor-alpha; ARG1: arginase 1; KGF-2: keratinocyte growth factor-2; RA: rheumatoid arthritis; PDGF: platelet-derived growth factor; Sp-1: specificity protein 1; PCNA: proliferating cell nuclear antigen; HMGA1: high mobility group A1; NR4A1: nuclear receptor subfamily 4 group A member 1; ZEB1: E-box binding homeobox 1; VGLL4: vestigial-like family member 4; CSI: cerebral ischemic injury; miR-93: microRNA-93; ATF4: activating transcription factor 4; PF11: pseudoginsenoside-F11; Bax: B-cell lymphoma 2 (BCL2) associated X; KLF2: Kruppel-like factor 2; JUNB: Jun B proto-oncogene; ETS1: E26 transformation-specific sequence (ETS) proto-oncogene 1; AD: Alzheimer's disease; BDNF: brain-derived neurotrophic factor; SCI: spinal cord injury; MMP: matrix metallopeptidase; mPR3: membrane proteinase 3; CSE: cystathionine-γ-lyase; AS: atherosclerosis; SLE: systemic lupus erythematosus; USP7: ubiquitin specific peptidase 7; HPK1: hematopoietic progenitor kinase 1; CREMα: cyclic adenosine monophosphate (cAMP) responsive element modulator alpha; Foxp3: forkhead box P3; CXCR3: C−X−C motif chemokine receptor 3; RORc: retinoic acid-binding receptor (RAR) related orphan receptor C; GATA3: GATA binding protein 3; RALDH: retinaldehyde dehydrogenases; PD: periodontal disease; GPNMB: glycoprotein nonmetastatic melanoma protein B; IRF4: IFN regulatory factor 4; SSc: systemic sclerosis; FRA2: Fos-related antigen 2; ADAM17: a disintegrin and metalloproteinase 17; IVL: involucrin; KRT1: keratin 1; FLG: filaggrin; LCE: late cornified envelope; GRHL2: grainyhead-like 2; RhoU: Ras homolog family member U; PLAU: plasminogen activator urokinase; β-MHC: beta-myosin heavy chain; CCND1: cyclin D1; COL1/3: type I/III collagen; CKD: chronic kidney disease.

### 4.1 The role of JMJD3 in lung inflammation

Acute lung injury (ALI) is a severe and potentially life-threatening condition characterized by an excessive inflammatory response, increased permeability of the alveolar membrane, and accumulation of pulmonary edema in the lungs. If left untreated, ALI may advance to acute respiratory distress syndrome, leading to mortality [110,111]. Bacterium-secreted LPS has the potential to induce ALI [35]. Neutrophils, Mфs, and endothelial cells play crucial roles in modulating inflammatory responses and inducing loss of lung vascular barrier functions in LPS-caused ALI [112]. During the LPS stimulation, JMJD3 expression was regulated by TLR4-NF-κB signaling [35], MAPK signaling, and STAT signaling [113]. Once regulated, JMJD3 promotes lung inflammation development by promoting the expressions of alarmins S100 calcium binding protein A8 (S100A8)/A9 [81], adenosine A2A receptor (ADORA2A), and C/EBPβ [80], and suppressing the expressions of nuclear factor erythroid-2-related factor-2 (Nrf2) [114] and IL-4i1 [35].

In other lung diseases, JMJD3 has been found to have both pro-inflammatory and anti-inflammatory effects depending on the context and cellular environment. In lung injury caused by ischemia-reperfusion, JMJD3 displayed pro-inflammatory effects by suppressing the expression of keratinocyte growth factor-2 (KGF-2) [79]. Conversely, in influenza infection-induced lung inflammation, JMJD3 exerted anti-inflammatory activity by elevating ARG1 levels. This is likely due to ARG1's ability to modulate the immune response and limit tissue damage caused by inflammation [68]. Moreover, AP can lead to inflammatory disorders of remote organs, such as lung injury, via the STING/TLR9-NF-κB-JMJD3-TNF-α pathway [34]. In summary, JMJD3 generally exerts pro-inflammatory effects in the lungs, but it can also exhibit anti-inflammatory effects through the IL-4/STAT6/IRF4 signaling pathway (Table 2 [34,35,68,[79], [80], [81]] and Fig. 3A).

### 4.2 The role of JMJD3 in RA and OA

RA is an autoimmune condition distinguished by persistent synovitis with cell proliferation, the infiltration of inflammatory cells into the synovial tissue of joints, and consequent cartilage degradation [115]. In RA, platelet-derived growth factor (PDGF) raises JMJD3 levels through the protein kinase B (Akt) and MAPK signaling pathways. The resulting upregulation of JMJD3 leads to a reduction in H3K27me3 in the promoter region of PCNA, ultimately leading to the increased transcription of PCNA. Elevated PCNA transcription is believed to contribute to the aggressive phenotype observed in RA [49]. Furthermore, it has been demonstrated that IL-1β can elevate JMJD3 protein levels via MAPK signaling and the transcription factor Sp-1. This upregulation leads to the removal of the H3K27me3 marker on the promoter of TLR2, thereby exacerbating the progression of RA [50].

OA is a prevalent chronic degenerative joint disease characterized by persistent joint pain and physical disability [116]. Despite the multifaceted nature of the OA pathology, including cartilage degradation, osteophyte formation, and subchondral bone sclerosis, the specific mechanisms underlying these changes remain a topic of ongoing debate [117]. In OA cartilage, upregulation of JMJD3 has been observed, with consequent activation of the NF-κB signaling pathway, which aggravates the destabilization of the medial meniscus (DMM)-induced cartilage damage *in vivo* [29]. In a separate study, increased JMJD3 levels in OA promoted disease progression by demethylating H3K27me3 at the nuclear receptor subfamily 4 group A member 1 (*NR4A1I*) promoter, thereby raising its levels. NR4A1 mediates pathological changes in chondrocytes by targeting the Akt pathway [83]. IL-1β has been found to induce oxidative stress injury and inflammation in chondrocytes, while also increasing the expression of high mobility group A1 (HMGA1), which binds to the promoter region of *JMJD3* to enhance its expression. Consequently, JMJD3 further stimulates the expression of E-box binding homeobox 1 (ZEB1) through its H3K27me3 demethylase activity. This is deleterious to chondrocytes, as ZEB1 has been implicated in promoting chondrocyte apoptosis and inhibiting extracellular matrix (ECM) synthesis [82]. In summary, JMJD3 consistently exhibited pro-inflammatory effects in the bones (Table 2 [49,50,82,83] and Fig. 3B).

### 4.3 The role of JMJD3 in neuroinflammation

Depression is a complex mental disorder that presents a range of symptoms, including feelings of sadness, loss of interest, reduced motivation, sleep problems, appetite changes, and cognitive difficulties [31,118]. Multiple studies have revealed a correlation between depression and the neuroinflammatory responses [119]. Cytokines, as key players in neuroinflammation, can impact behaviors by altering neurochemical processes in the brain. Previous studies have displayed that administering LPS increases the levels of pro-inflammatory cytokines, such as IL-6, TNF-α, and IL-1β, and induces depression-like behaviors in mice [32]. CUMS during adolescence induces depression-like behaviors in rats and alters epigenetic regulation in regions, such as the PFC and HIP. Specifically, CUMS leads to increased expression of pro-inflammatory cytokines, some of which are pro-inflammatory molecules involved in the immune response. This was accompanied by increased JMJD3 and decreased H3K27me3 expression, indicating disrupted gene expression patterns [33]. Another study demonstrated that JMJD3 contributes to microglial activation, leading to the increased expression of pro-inflammatory cytokines in the PFC and HIP, ultimately causing depressive disorders. This study also found that a decrease in adiponectin levels reduced the binding between HMGA1 and H3K27me3, resulting in excessive demethylation of H3K27me3 and further contributing to the development of depression [31]. Furthermore, dehydroepiandrosterone (DHEA) reduces microglia-mediated inflammation in a mouse model of acute LPS-induced neuroinflammation and in cultured microglia *in vitro*. DHEA modulates the inflammatory response in microglia by phosphorylating tyrosine receptor kinase A (TrkA) and subsequently activating pathways involving Akt1/Akt2 and cyclic adenosine monophosphate (cAMP)-responsive element-binding proteins. The latter induces the expression of JMJD3, which controls the expression of inflammation-associated genes and microglial polarization [120]. However, opposing results were observed in a study examining depression-like behavior induced by maternal separation and upregulation of pro-inflammatory factors associated with the JMJD3 and NF-κB pathways as well as activation of microglia [32]. Under inflammatory conditions, activation of JMJD3 leads to increased VGLL4 activity in the HIP of mice, enhancing microglial activity and promoting IL-1β production via the STAT3 pathway, which leads to neuroinflammation and anxiety-like behavior [40]. Taken together, these findings suggest that JMJD3 and its associated inflammatory pathways play important roles in the development of depression and related behaviors.

Cerebral ischemic injury (CSI) is indeed a significant factor in inducing brain inflammation, and ischemic stroke is a prevalent neurological disorder that can lead to disability and mortality following cardiovascular events worldwide [121,122]. Oxygen-glucose deprivation (OGD) could induce the release of lactate dehydrogenase, indicating cytotoxic effects on astrocytes due to ischemia or anoxia. Furthermore, central nervous system (CNS) injury triggers reactive astrogliosis, activating astrocytes, and altering gene expression. Excessive astrogliosis released during later stages of CNS injury was found to contribute to neuronal insults [123]. In experimental models using OGD to simulate CSI, JMJD3 is upregulated in cultured neurons and it promotes neuronal cell death by regulating the levels of BCL2 associated X (Bax) and caspase-3, which are key players in the apoptotic pathway [73]. Furthermore, the JAK/STAT pathway has been found to regulate JMJD3 expression, which promotes astrocyte apoptosis in response to OGD injury [123]. Additionally, miR-93 secreted by bone marrow mesenchymal stem cells (BMSC)-extracellular vesicles (EVs) regulates the JMJD3/p53/Kruppel-like factor 2 (KLF2) axis, contributing to a reduction in OGD-induced hippocampal neuron apoptosis *in vitro*. miR-93 in BMSC-EVs can alleviate hypoxic-ischemic brain injury *in vivo* [77]. Another study demonstrated that miR-148a-3p plays a negative regulatory role by mediating STAT proteins to inactivate JMJD3. Overexpression of STAT1 and STAT3 was found to promote pyroptosis in astrocytes; however, this effect was counteracted by the overexpression of miR-148a-3p, suggesting its protective role against pyroptosis [124]. Activating transcription factor 4 (ATF4), a transcription factor regulating mitochondrial respiration, glycolysis, and amino acid metabolism, as well as CD4^+^ T cell proliferation and effector function [125], worsens CSI by upregulating Jun B proto-oncogene (JUNB) and E26 transformation-specific sequence (ETS) proto-oncogene 1 (ETS1) expression in a JMJD3-dependent mode [126]. Collectively, the above data supports that JMJD3 promotes the progression of neuroinflammation. Interestingly, there are two studies about the anti-inflammatory role of JMJD3 in neuroinflammation. One study demonstrated that pseudoginsenoside-F11 (PF11) exerted a protective effect on ischemic neurons by inducing the JMJD3-dependently polarization of M2-like microglia/Mфs. This mechanism contributes to the functional recovery observed after transient cerebral ischemia [75]. Furthermore, another study demonstrated that inhibiting EZH2 has a protective effect against ischemic brain injury by modulating the H3K27me3/PI3K/Akt/mechanistic target of rapamycin (mTOR) pathway. As JMJD3 is an antagonistic protein of EZH2, it is plausible that JMJD3 also plays a protective role against ischemic brain injury [127].

Alzheimer's disease (AD) is a neurodegenerative condition characterized by dementia and is the most common form of dementia globally. The incidence of AD is on the rise [128,129]. The aggregation of amyloid-beta protein (Aβ) and the subsequent generation of Aβ_1__–__40_ have been implicated in neuronal dysfunction and apoptosis, playing a pivotal role in AD progression [130]. Furthermore, Aβ aggregation has been associated with the activation of the mitochondrial stress response (MSR), which constitutes a pathological mechanism in AD. This activation leads to mitochondrial dysfunction by perturbing the normal functioning of mitochondrial proteins [131]. JMJD3 can improve the levels of brain-derived neurotrophic factor (BDNF) by inducing demethylation of H3K27me3, which helps maintain the balance of MSR, ultimately preventing the progression of AD [76,78]. However, JMJD3 also plays a pro-inflammatory role in the development of alcohol addiction [132], and astrocytic damage following cavitation [133]. Furthermore, following spinal cord injury (SCI), the blood-spinal cord barrier (BSCB) is disrupted, leading to secondary injury characterized by apoptotic cell death of neurons and oligodendrocytes, ultimately resulting in permanent neurological deficits. However, JMJD3-mediated H3K27me3 demethylation regulates the inflammatory response and maintains BSCB integrity following SCI [134]. Additionally, upregulated JMJD3 and NF-κB signaling associated with proteins promote *MMP-3* and *MMP-9* gene expression after SCI in injured vascular endothelial cells [72,74]. In summary, JMJD3 plays a dual role in brain-related inflammation (Table 2 [40,61,[72], [73], [74], [75], [76], [77], [78]] and Fig. 3C).

### 4.4 The role of JMJD3 in sepsis

Sepsis is a severe medical condition characterized by life-threatening organ dysfunction. This arises from the overwhelming activation of the host's immunity to infection [85,135,136]. The inflammatory environment of sepsis involves an exaggerated inflammatory response characterized by producing pro-inflammatory cytokines. Neutrophils play a crucial role in the innate immune response, exhibiting reverse migration and interacting with other immune cells through membrane-associated proteins. Additionally, the neutrophil membrane proteinase 3 (mPR3) demonstrates pro-inflammatory properties and unique substrate specificity, contributing to the pathogenesis of early sepsis [88]. When a host encounters a pathogen, the innate immune system recognizes the pathogen-associated molecular patterns and triggers an immune response. This response includes the release of pro-inflammatory cytokines like IL-1β. However, excessive and uncontrolled release of these cytokines can lead to a cytokine storm characterized by an intense inflammatory response. Cytokine storms can further amplify inflammatory reactions, leading to multiple organ failure and an increased risk of early mortality in patients with sepsis [137,138]. After LPS stimulation, the MAPK signaling pathway is activated, which in turn downregulates the STAT signaling pathway and promotes the expression of JMJD3 [113]. The upregulation of JMJD3 has been shown to promote the expression of mPR3 and IL-1β, leading to a pro-inflammatory response [88]. Notably, certain factors (e.g., cystathionine-γ-lyase (CSE)/H_2_S [108], miR-146a [27], and miR-27b [28]) have been found to inhibit the inflammatory response mediated by the JMJD3 and NF-κB signaling pathways, thus providing protection to septic patients. In doing so, they may help mitigate excessive inflammation associated with sepsis and potentially contribute to patient protection.

### 4.5 The role of JMJD3 in AS

AS is commonly linked to cardiovascular and cerebrovascular diseases [139]. Traditionally, AS is understood as a chronic inflammatory condition affecting the arterial wall due to irregular lipid metabolism. Monocytes and Mфs are implicated in starting and advancing atherosclerotic lesions [140]. However, new drivers of AS have emerged, including noise, air pollution, disturbed sleep, age, and age-related clonal hematopoiesis of indeterminate potential [141,142]. Serum amyloid A (SAA) is a pro-inflammatory protein associated with AS pathogenesis of ankylosing spondylitis, and it induces the elevation of JMJD3 in Mфs and thus reduces H3K27me3 levels. The knockdown or inactivation of JMJD3 results in impaired expression of pro-inflammatory genes associated with the NF-κB pathway in response to SAA stimulation. This impairment is linked to the restoration of H3K27me3 levels [26,[143], [144], [145]]. Notably, the deletion of JMJD3 has been associated with advanced AS, indicating a complex role of JMJD3 in cardiovascular health [140]. Further exploration is imperative to fully understand the role of JMJD3 in the regulation of SAA-induced AS. Additionally, in AAAs, a condition characterized by similar inflammation to AS, it has been observed that IFN-β regulates JMJD3 expression through the JAK/STAT pathway. Furthermore, JMJD3 has been found to induce NF-κB-mediated transcription of inflammatory genes in infiltrating aortic Mфs, thereby promoting AAA expansion [30]. These findings highlight the multifaceted role of JMJD3 in modulating inflammatory responses and its potential implications in various inflammatory conditions including AS and AAAs.

### 4.6 The role of JMJD3 in systemic lupus erythematosus (SLE)

SLE is a complex chronic autoimmune disease characterized by dysregulation of the immune system, leading to damage in multiple organs or systems. Dysfunction in immune activity, including abnormalities of the innate immune response, autoreactive T cells, and overactive B cells, contributes to the pathogenesis of SLE [146]. Hematopoietic progenitor kinase 1 (HPK1) acts as an inhibitor of T cells [89], and JMJD3 has been identified to regulate HPK1's expression. In CD4^+^ T cells of individuals with SLE, the reduction of JMJD3 binding to the *HPK1* promoter region leads to an enrichment of H3K27me3 and subsequent downregulation of HPK1 expression. This abnormality in T cell reactivity ultimately contributes to autoimmunity [84,89]. Furthermore, JMJD3 has been found to raise the levels of CD11a and cAMP responsive element modulator alpha (CREMα) in CD4^+^ T cells, further promoting the progression of SLE [86,87]. Ubiquitin specific peptidase 7 (USP7) stabilizes JMJD3 via deubiquitylation, leading to increased pro-inflammatory gene expression related to the NF-κB pathway and lupus nephritis progression [147]. Moreover, low p53 expression has been linked to reduced miR-1246 levels in B cells of patients with SLE [148]. In summary, JMJD3 plays a pro-inflammatory role in blood-related inflammation; however, its absence results in more advanced AS. However, the specific mechanisms underlying this phenomenon require further investigation (Table 2 [27,28,30,[84], [85], [86], [87], [88], [89]] and Fig. 3D).

### 4.7 The role of JMJD3 in intestine inflammation

JMJD3 plays a role in intestinal inflammation, particularly in various forms of colitis such as necrotizing enterocolitis and ulcerative colitis (UC). These conditions are characterized by recurring abdominal pain, mucosanguineous feces, diarrhea, and tenesmus. It is widely acknowledged that colitis triggers an immune response in the intestinal mucosa, particularly in genetically susceptible individuals due to a combination of environmental factors and intestinal flora. This immune response sets off a series of inflammatory reactions, causing an imbalance in anti-inflammatory and pro-inflammatory mediators and resulting in pathological alterations in the intestinal mucosa. Thus, JMJD3 may be involved in modulating inflammatory responses during intestinal inflammation [149]. JMJD3-mediated activation of the T-bet pathway results in the upregulation of key differentiation factors for Th1 and Treg cells, including forkhead box P3 (Foxp3), CD44, and C–X–C motif chemokine receptor 3 (CXCR3), while concurrently downregulating factors linked to Th2 cell differentiation such as retinoic acid-binding receptor (RAR) related orphan receptor C (RORc) and GATA3. This dual effect promotes the differentiation of Th1 and Treg cells while inhibiting the differentiation of Th2 and Th17 cells [47]. Additionally, JMJD3 has been observed to directly bind to genomic sites associated with the master Th17 transcription factor RORγt and Th17 cytokine genes, leading to a reduction in H3K27me3 levels. This finding suggests that JMJD3 suppresses Th17 cell differentiation and the expression of Th17-related genes by modulating H3K27me3 levels, particularly in the spleen and lymph nodes [90]. These findings underscore the complex and multifaceted role of JMJD3 in orchestrating the differentiation of distinct Th cell subsets. JMJD3 has been found to regulate intestinal inflammation, particularly during the progression of colitis. It activates the NLRP3 inflammasome by reducing the enrichment of H3K27me3 in the *Nrf2*'s promoter, resulting in elevating Nrf2 levels and subsequent promotion of NLRP3 inflammasome assembly, ultimately contributing to colitis progression [91]. Additionally, JMJD3 levels have been found to be raised in the colonic mucosa of patients with active UC and negatively associated with both vitamin D receptor expression and H3K27me3 levels, suggesting a potential function in the progression of UC [92]. JMJD3 has also been shown to inhibit the activity of retinaldehyde dehydrogenases (RALDH) and promote the NF-κB and JAK2/STAT3 pathways, contributing to the progression of colitis [41,42,150]. In summary, it is involved in the regulation of intestinal inflammation by raising the levels of pro-inflammatory cytokines and regulating the differentiation of Th cells (Table 2 [47,[90], [91], [92]] and Fig. 3E).

### 4.8 The role of JMJD3 in periodontal disease (PD)

PD is a prevalent chronic oral condition that is primarily characterized by inflammation of the gums, periodontal pocket formation, alveolar bone resorption, and tooth displacement or loss [39]. The inflammatory environment in periodontitis is driven by dysbiosis of the bacterial biofilm and an imbalance between pro-inflammatory/anti-inflammatory cytokines. Monocytes and Mфs play crucial roles, with M1-like Mфs promoting early inflammation and M2-like Mфs driving resolution and tissue repair. This interplay contributes to the pathogenesis of periodontitis [151]. Two key events are observed in PD: inflammation and alveolar bone resorption/tooth loss. Inflammatory events include gingivitis and periodontitis [[152], [153], [154]]. Gao et al. [108] found that JMJD3 regulates the regulation of *IL-6* and *IL-12* activation in hPDLs by demethylating H3K27me3 in their promoters. Furthermore, JMJD3 and pro-inflammatory factors associated with NF-κB were found to be upregulated by LPS in periodontal ligament cells [95]. Furthermore, under LPS stimulation, JMJD3 facilitates the differentiation of Th17 cells via the STAT3-RORc pathway, which is implicated in the differentiation and function of Th17 cells. By affecting this pathway, JMJD3 promotes the generation of Th17 cells [94]. Although it promotes the progression of PD, it can also inhibit dental inflammation. Adiponectin has been identified as a modulator that mitigates periodontal bone loss by modulating the JMJD3-IRF4 axis, which is crucial for the transformation of M2-like Mфs [93]. The protein GPNMB demonstrates elevated expression levels in gum tissue affected by PD and seems to function as an anti-inflammatory factor in PD development by influencing JMJD3 through both the TLR4-IκB pathway and the STAT3 pathway [39]. Additionally, individuals with both type 2 diabetes (T2D) and PD exhibit increased levels of the pro-inflammatory transcription factors STAT1 and IRF1 along with a decrease in JMJD3 expression in circulating monocytes [151]. In summary, JMJD3 plays a dual role in the modulation of dental inflammation (Table 2 [[93], [94], [95],^108^] and Fig. 3F).

### 4.9 The role of JMJD3 in skin inflammation

There are a few studies about the role of JMJD3 in skin inflammation. In pathological skin inflammation, JMJD3 promotes the progression of SSc by modulating the occupations of H3K27me3 at *FRA2*'s promoter. This regulation leads to the activation of fibroblasts in a TGF-β-dependent manner [57]. JMJD3 has also been implicated in promoting inflammation in endothelial cells by regulating the activation of the metalloproteinase a disintegrin and metalloproteinase 17 (ADAM17) and Notch ligand Jagged-1, thereby activating the Notch pathway [99]. However, in hyperproliferative skin diseases, JMJD3 appears to exhibit an anti-inflammatory role, as evidenced by the downregulation of expression of grainyhead-like 2 (GRHL2), which in turn inhibits the levels of epidermal differentiation complex (EDC) genes such as filaggrin (*FLG*), involucrin (*IVL*), late cornified envelope (*LCE*s), keratin 1 (*KRT1*), and small proline-rich proteins (*SPRR*s), thereby promoting the progression of hyperproliferative skin diseases [98]. Conversely, in allergic contact dermatitis (ACD), JMJD3 has been found to neither promote nor inhibit inflammatory progression. UTX, another member of the KDM6 family, plays a pro-inflammatory role in ACD. Notably, the specific roles of JMJD3 and UTX in ACD may vary depending on the specific molecular mechanisms involved, thus necessitating further research to comprehensively understand their contributions to this particular skin condition [155].

Furthermore, the role of JMJD3 in traumatic skin inflammation varies depending on the context. Tissue repair after injury involves a series of coordinated stages including coagulation, inflammation, proliferation, and remodeling [156,157]. The inflammatory phase can be separated into two stages: an initial stage, where Mфs support inflammation and pathogen destruction, and a late phase, where they facilitate tissue repair [158]. Under normal conditions, both JMJD3's histone demethylation activity and NF-κB signaling are essential for wound healing in keratinocytes [159,160]. Both signaling could upregulate notch receptor 1 (Notch1) levels and thus lead to raising the levels of Ras homolog family member U (*RhoU*) and plasminogen activator urokinase (*PLAU*) genes, which are vital regulators of cell migration [107]. However, in T2D, elevated levels of palmitate have been observed, which in turn induces the upregulation of JMJD3 in Mфs through a TLR4/myeloid differentiation primary response protein 88 (MyD88)-dependent pathway. Subsequently, JMJD3-dependent epigenetic modifications occur, leading to a pro-inflammatory state characterized by removing inhibitory histone methylation marks on the promoters of inflammatory cytokines. These processes enhance impaired wound healing in diabetic wounds in individuals with diabetes [97]. Additionally, we found that IL-6 regulates JMJD3 expression in Mфs through the JAK1/STAT3 pathway and that this increase in JMJD3 expression induces the transcription of NF-κB-mediated inflammatory genes in Mфs through the H3K27me3 mechanism [98]. Notably, miR-106b, derived from EVs released by HUVECs, inhibits the expression of JMJD3 and receptor-interacting protein kinase 3 (RIPK3), thereby impairing skin wound healing [161]. These findings indicate that while JMJD3 is crucial for normal wound healing, its excessive promotion of inflammation can inhibit proper wound healing in the presence of underlying diseases, such as diabetes (Table 2 [57,[96], [97], [98], [99],^107^] and Fig. 3G).

### 4.10 The role of JMJD3 in muscle inflammation

Previous investigations of JMJD3 and muscle-related inflammation have mainly focused on heart diseases, such as cardiac hypertrophy [100], myocardial infarction [162,163], and myocardial fibrosis [101]. In the context of cardiac hypertrophy, JMJD3 raises beta-myosin heavy chain (β-MHC) levels by demethylating H3K27me3 at β-MHC's promoter, thereby contributing to cardiac hypertrophy [100]. Doxorubicin-induced cardiomyopathy is characterized by negative regulation of Sestrin2 expression by JMJD3, which reduces the enrichment of H3K27me3 in the *Sestrin2*'s promoter region. Downregulation of Sestrin2 leads to mitochondrial dysfunction and cardiomyocyte apoptosis. The impact of JMJD3 on Sestrin2 expression highlights its role in modulating cellular responses to cardiotoxic stress and suggests that it could be an important therapeutic target for addressing doxorubicin-induced cardiomyopathy [102]. In ischemic heart disease, the activation of the TGF-β pathway has been implicated as a potential impediment to the reprogramming of fibroblasts into cardiomyocytes due to its tendency to interfere with GATA4-JMJD3 interactions. This interference results in a decrease in the expression of genes specific to cardiac cells and a reduction in the number of reprogrammed cardiomyocytes [102]. In addition to its involvement in myocardial inflammation, JMJD3 is associated with muscle inflammation [101]. Specifically, JMJD3 has been shown to decrease the occupations of H3K27me3 at the *β-catenin* promoter in activated cardiac fibroblasts, raising the expression of genes related to fibrosis and ultimately contributing to the advancement of myocardial fibrosis. Following a myocardial infarction, JMJD3 stimulates the expression of pro-inflammatory cytokines, potentially contributing to the onset of depression [162,163]. Studies of muscle inflammation, apart from myocardial inflammation, are limited. However, a particular research study on human bladder smooth muscle cells revealed that LPS stimulation significantly activated the NF-κB pathway and led to elevating JMJD3 levels. Activating NF-κB-JMJD3 signaling was found to significantly facilitate cell proliferation and collagen accumulation by raising the expression of cyclin D1 (CCND1) and type I/III collagen (COL1/3), respectively. Promotion of bladder detrusor hyperplasia and reduced compliance could contribute to disease progression [109]. Furthermore, JMJD3 has been implicated in facilitating muscle repair processes through its involvement in hyaluronic acid synthesis, potentially exerting a proregenerative effect [164]. In summary, JMJD3 has generally been associated with pro-inflammatory effects in muscle tissues, most notably in the heart. However, its role is multidimensional and includes potential pro-regenerative functions. Further research is required to comprehensively understand the intricate mechanisms of JMJD3 in muscle inflammation and to explore its possible therapeutic implications (Table 2 [[100], [101], [102],^109^] and Fig. 3H).

### 4.11 The role of JMJD3 in kidney inflammation

Tubulointerstitial fibrosis is recognized as the primary pathological process contributing to the progression from chronic kidney disease (CKD) to end-stage renal disease (ESRD) [103,104]. This process is dynamic and characterized by the activation of renal interstitial fibroblasts and excessive accumulation of ECM proteins, such as collagens I and III, within the renal interstitium [[165], [166], [167]]. Following stress, Th2 cytokines like IL-4 can stimulate the JMJD3/IRF4 axis, triggering the activation of myeloid fibroblast, transition to myofibroblasts, and ultimately promoting kidney fibrogenesis [[104], [105], [106]]. Conversely, an alternative study indicated that JMJD3 exerts anti-fibrotic activity by promoting the degradation of Notch1 and Notch3, while concurrently inhibiting the activation of the Akt and ERK1/2 pathways [103]. DN is a microvascular complication linked to diabetes and constitutes the leading cause of mortality in individuals with type 1 diabetes mellitus, as well as the second most common cause of mortality in those with T2D. DN is also recognized as the primary reason for ESRD [168,169]. In summary, JMJD3 plays a dual role in kidney inflammation and further research is essential to elucidate its specific mechanisms (Table 2 [[103], [104], [105], [106]] and Fig. 3I).

## 5 Role of JMJD3 in the treatment of inflammation

Although there has been a growing understanding of the role of JMJD3, selective JMJD3 inhibitors remain a matter of concern. Given the substantial involvement of JMJD3 in various human diseases, it is essential to develop inhibitors that target JMJD3 as potential therapeutic agents for treating these conditions (Table 3 [18,[170], [171], [172], [173], [174], [175]]). Based on their structural properties, JMJD3 inhibitors are classified into the following five subcategories:

**Table 3:** The main characteristics of Jumonji domain-containing protein D3 (JMJD3) inhibitors.

Compound No.	Structure	Chemical library (number)	IC_50_ (μM)	Screening methods	Mechanism	Refs.
**1**		∼2,000,000	0.06	AlphaScreen	Competitive inhibitor	[18]
**2**		–	>100	AlphaScreen	–	[18]
**3**		–	–	–	–	[18]
**4**		–	>50	Mass spectrometry	–	[18]
**5**		–	>50	Mass spectrometry	–	[18]
**6**		18	0.2	AlphaLISA	Competitive inhibitor	[170]
**7**		18	0.15	AlphaLISA	Competitive inhibitor	[170]
**8**		18	0.27	AlphaLISA	Competitive inhibitor	[170]
**9**		18	0.21	AlphaLISA	Competitive inhibitor	[170]
**10**		52	1.2	AlphaScreen	Noncompetitive inhibitor	[171]
**11**		8	8.34	Dose-response assays	Competitive inhibitor	[172]
**12**		8	20.31	Dose-response assays	Competitive inhibitor	[172]
**13**		8	–	–	–	[172]
**14**		8	–	–	–	[172]
**15**		68	1.52	AlphaScreen	Competitive inhibitor	[173]
**16**		68	–	AlphaScreen	Competitive inhibitor	[173]
**17**		68	–	AlphaScreen	Competitive inhibitor	[173]
**18**		22,545	12	AlphaScreen	Noncompetitive inhibitor	[174]
**19**		500,000	7.64	AlphaLISA	Noncompetitive inhibitor	[175]
**20**		500,000	2.99	AlphaLISA	Noncompetitive inhibitor	[175]
**21**		500,000	6.27	AlphaLISA	Noncompetitive inhibitor	[175]

–: no data; IC_50_: half maximal inhibitory concentration.

### 5.1 2-OG analogs

**Fig. 4:**
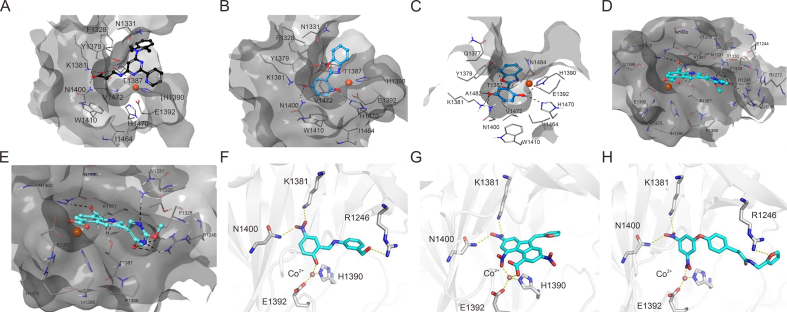
The docking analysis of Jumonji domain-containing protein D3 (JMJD3) with some of its inhibitors. (A) Compound **1**-4ASK [171]. (B, C) Compound **10**: 4ASK **(**B) and 2XXZ (C) [171]. (D, E) Compound **15**: 4ASK (D) and 2XXZ (E) [173]. (F) Compound **19** [175]. (G) Compound **20** [175]. (H) Compound **21** [175]. 4ASK and 2XXZ are two X-ray structures of JMJD3 in the Research Collaboratory for Structural Bioinformatics (RCSB) Protein Data Bank (PDB) (https://www.rcsb.org/). Reprinted from Refs. [171,173,175] with permission.

The development of potent and competitive JMJD3 inhibitors has been significantly advanced by targeting 2-OG analogs, given 2-OG's essential role as a cofactor for the demethylase activity of JmjC-KDMs, including JMJD3. This approach leverages the critical interaction between 2-OG and the enzyme to design molecules that mimic 2-OG's binding but inhibit the enzyme's function [8]. Compound **1** (Table 3 [18]), known as GSK-J1, emerged as the first identified inhibitor specifically targeting JMJD3. It mimics the binding of 2-OG by maintaining interactions with key residues K1381, T1387, and N1480 within JMJD3. Compound **1** impressively inhibited JMJD3 activation with a half maximal inhibitory concentration (IC_50_) value of 0.06 μM, as determined by an AlphaScreen assay. A notable feature of compound **1** is its pyridyl-pyrimidine biaryl structure, which forms a bidentate interaction with the catalytic metal ion, leading to a significant 2.34 Å shift of the Fe^2+^ ion away from the conserved histidine-histidine-glutamate triad crucial for enzyme activity. This bidentate interaction is pivotal for the inhibition mechanism of compound **1**. In contrast, the pyridine regio-isomer of compound **1**, compound **2**, lacks the ability to form such bidentate interactions, resulting in considerably weaker JMJD3 inhibition (IC_50_ > 100 μM), which highlights the importance of specific structural features for effective enzyme inhibition [170]. The co-crystal structure of compound **1** with JMJD3 (Fig. 4A) [171] revealed that strategic substitution possibilities at the *para* position to the pyridine nitrogen, facilitating solvent access and enabling modifications such as immobilization on beads, leading to the creation of compound **3**, an amine analog of **1** used for bead immobilization studies. The highly polar carboxylate group of compound **1** is critical for *in vitro* binding to JMJD3. However, the highly polar carboxylate group of 1, while critical for *in vitro* binding to JMJD3, poses challenges for cellular permeability. To address this, the polarity of the acid groups in compounds **1** and **2** was masked with ethyl esters, yielding compounds **4** and **5**. These cell-penetrating esters exhibit weaker *in vitro* IC_50_ values (>50 μM) but are rapidly hydrolyzed by esterases in Mфs to release the active inhibitors within cells, achieving pharmacologically relevant concentrations. Ethyl ester pro-drug, compound **4,** demonstrated cellular activity in flag-JMJD3-transfected HeLa cells by preventing the JMJD3-induced loss of nuclear H3K27me3 immunostaining. Moreover, administration of compound **4** increased total nuclear H3K27me3 levels in untransfected cells, showcasing its potential therapeutic relevance by modulating histone methylation states within cells. This strategic approach of modifying 2-OG analogs to develop JMJD3 inhibitors exemplifies the intricate balance between molecular design for target specificity and modifications for enhanced cellular delivery and activity [18]. Through modifications to the structure of compound **1**, compounds **6**–**9** (Table 3 [170]) were developed, with compound **6** maintaining the key pharmacophores of beta amino acid and benzoazepine. The modification involved changing the chelating scaffold to 2-(pyrimidine-4-yl)thiazole. In contrast, compounds **7**–**9** replaced this thiazole with pyrazoles and two different triazoles, exploring alternative heterocyclic ring effects on JMJD3 inhibition. Among these, compound **6** exhibited superior efficacy compared to compound **1** and showed slightly better activity than the triazole-based compound **5**. The IC_50_ values for compounds **6**–**9** against JMJD3 activation were 0.2, 0.15, 0.27, and 0.21 μM, respectively, as determined by the AlphaLISA immunodetection assay. Their ethyl ester forms significantly inhibited LPS-induced immune responses, demonstrating high cellular activity at a concentration of 0.82 μM [170]. Compound **10** (Table 3 [171]), synthesized via microwave-assisted coupling of the carboxylic acid with 2-hydroxy-aminophenol under catalyst- and solvent-free conditions, induced cell cycle arrest in A375 cancer cells. It specifically targeted KDM6 histone demethylases, showing IC_50_ values of 25.7 μM against UTX and 1.2 μM against JMJD3, as measured by the AlphaScreen assay. The co-crystal structure of compound **10** with JMJD3 (Figs. 4B and C) [171] revealed that its benzoxazole portion engaged in π−π interactions with H1390's side chain, akin to compound **1**, and formed hydrogen bonds with Y1379. Additionally, the phenol moiety of compound **10** also participated in π−π interactions with Y1381, while its hydroxyl group established hydrogen bonds with H1470. The C-4 hydroxyl group on the phenol ring similarly formed hydrogen bonds with K1381, T1387, and N1400. Moreover, Fe^2+^ ions were bidentately coordinated by the nitrogen and hydroxyl groups of compound **10**, with the oxygen of its five-membered ring hydrogen-bonding to Y1379, further enhancing its interaction with the enzyme [171].

### 5.2 Metal-based inhibitors

Kinetically inert transition metal complexes have garnered substantial attention in the field of pharmaceutical discovery. These complexes have attracted considerable interest because of their exceptional properties, including stability, tunability, catalytic activity, high binding affinity, and versatility. The unique characteristics of these complexes make them highly desirable for diverse pharmaceutical applications and have led to extensive research in this field [[176], [177], [178], [179]]. Rhodium (III)-based metal complexes have significant inhibitory effects on KDMs, such as KDM5A [4,180], LSD1 [181], and JMJD2 [182]. Kang et al. [172] also identified a rhodium-based metal complex (compound **11**) with 2 C−N ligands 1-phenylisoquinoline and 1 N−N ligand 4,4′-dinonyl-2,2′-bipyridine as a JMJD3 inhibitor (Table 3 [172]). In the mechanism, compound **11** could block the protein-protein interactions (PPI) between JMJD3 and H3K27me3 and thus lead to the accumulation of H3K27me3, which reduced TNF-α level by targeting its transcription start site. Compound **12**, an iridium (III) analog of compound **11**, was identified in prior research as an antagonist of JMJD2D, demonstrating significant inhibitory activity with an IC_50_ value of 8.34 μM. This makes compound **11** notably more potent by over twice the efficacy, compared to its counterpart, compound **12**, which showed an IC_50_ value of 20.31 μM. Furthermore, compound **11** was found to suppress the proliferation of RAW264.7 cells, a Mф cell line, with an IC_50_ value of 15.02 ± 1.42 μM, while compound **12,** with the same N−C and N−N ligands but different metal center with compound **11**, displayed more potent against RAW264.7 cells with IC_50_ value of 4.5 ± 1.17 μM. The comparison between the rhodium(III) compound **11** and its iridium(III) analog compound **12** highlights the significant impact that the central metal ion can have on the biological activity of these complexes, with rhodium(III) proving to be more effective in this context. Further investigation into compounds **13** and **14**, which share the same 4,4′-dinonyl-2,2′-bipyridine N−N ligands as compounds **11** and **12** but differ by incorporating smaller 2-phenylpyridine C−N ligands instead of the 1-phenylisoquinoline ligands found in compound **11**, reveals an interesting trend. Both compounds **13** and **14** displayed enhanced inhibitory activity against JMJD3 compared to their predecessors. This observation suggests that the presence of extended C−N ligands contributes positively to the inhibition of JMJD3, indicating that the ligand structure, particularly the size and nature of the C−N ligands, plays a crucial role in determining the inhibitory potency of these metal complexes [172]. This insight into the structure-activity relationship underscores the importance of both the metal center and ligand architecture in designing effective inhibitors against specific targets like JMJD3, offering valuable guidance for future drug development endeavors.

### 5.3 Quinolines

Quinoline derivatives are recognized for their biological inhibitory capabilities, which include competing with 2-OG, engaging Fe^2+^ at the active sites, and facilitating metal translocating [183]. These properties have led to their extensive applications in creating inhibitors for KDMs, notably KDM4A [184] and KDM6A [8]. A fragment-based strategy pinpointed a quinoline-5,8 dicarboxylic acid scaffold as an effective lead for crafting JMJD3 inhibitors, making a step forward in therapeutic development [173]. Compound **15** (Table 3 [173]), distinguished by its ability to inhibit JMJD3 activation with an IC_50_ value of 1.52 μM, demonstrates specificity in its action, as evidenced by its co-crystal structure with JMJD3 (Figs. 4D and E) [173]. In contrast to compound **1**, compound **15** shows negligible inhibition of UTX at a 10 μM concentration. Mechanistically, compound **15** exhibits a distinct mechanism of interaction with the Fe^2+^ ion within the catalytic site, engaging in bidentate coordination through its carboxylate group at the C5 position. Additionally, its second carboxylic functionality engages in ionic interactions with residue K1381 and forms hydrogen bonds with T1387 and N1400. These specific interactions mirror those observed in the co-crystal structure of the inhibitor (compound **1**), highlighting a common binding motif among effective JMJD3 inhibitors. The quinoline moiety of compound **15** further contributes to its inhibitory activity through π−π stacking interactions with Y1379, while also establishing a hydrogen bond with T1387. This multifaceted engagement underscores the importance of aromatic systems in mediating strong interactions within the catalytic pocket of JMJD3. In contrast, compound **3** exhibits variation in the nature of interactions, particularly due to the substituents at the C3 position. For instance, the methoxy group located at the C2 position on the pyrimidine ring of compound **15** forms hydrogen bonds with R1246 and N1331, enhancing its binding affinity. Moreover, the nitrogen atom at position 1 of the same ring also engages in a hydrogen bond with N1331, while the pyrimidine ring itself interacts with N1246, demonstrating the compound's ability to form multiple points of contact within the enzyme's active site. The presence of a second methoxy group in compound **15** facilitates van der Waals interactions with the side chains of F1328, T1387, and P1388, further stabilizing the compound within the catalytic pocket. Compounds **16** and **17**, bearing sulfonamide and carboxylic acid groups respectively, are noted for accepting two hydrogen bonds from R1246 and N1331, similar to compound **15** [173]. However, despite forming π−cation interactions with R1246, these compounds fail to orient their functional groups in a manner that would allow for optimal interaction with R1246, suggesting a limitation in their binding efficacy. This detailed analysis of the binding interactions of compounds **15**–**17** within the JMJD3 active site reveals the complexity of molecular recognition and the significance of precise structural features in dictating the potency and specificity of enzyme inhibitors. It underscores the potential for designing more effective inhibitors by carefully considering the orientation and nature of functional groups to enhance binding interactions within the target enzyme's active site.

### 5.4 Pyridines

Pyridine derivatives have emerged as highly potent inhibitors against various KDMs due to their ability to chelate metal ions within active sites of histone demethylases through their pyridine rings. This chelating action is central to their mechanism of inhibition, making the pyridine moiety a key component in the development of KDM inhibitors. As a result, pyridine-based compounds have been extensively utilized in crafting inhibitors for several KDMs, including KDM4A [184], KDM6A [8], and JMJD3. Among these derivatives, compound **18**, depicted in Table 3 [174], showcased significant inhibitory activity towards JMJD3, with an IC_50_ value of 12 μM. The inhibition mechanism of compound **18** involves strategic interactions within the catalytic domain of JMJD3. Specifically, the N-methyl substituent of compound **18** extends into a hydrophobic subpocket, which is delineated by the side chains of residues Y1379 and A1482 in JMJD3. This positioning allows for optimal orientation and interaction with the enzyme. Furthermore, compound **18** engages in multiple bonding interactions that contribute to its inhibitory effect. The carboxyl group of compound **18** participates in electrostatic interactions with residue K1381, forming direct hydrogen bonds with T1387, N1400, and N1480. Additionally, it establishes water-mediated hydrogen bonding with the backbone carboxyl oxygen of S1385. These interactions highlight the compound's precise and multifaceted binding mechanism, underscoring the significance of structural design in developing effective KDM inhibitors. Through such detailed molecular engagement, compound **18** effectively inhibits the activation of JMJD3, demonstrating the potential of pyridine derivatives in therapeutic applications targeting histone demethylases [174].

### 5.5 Others

Compounds **19**–**21** (Table 3 [175]) demonstrated promising activity, with the IC_50_ values of 7.64, 2.99, and 6.27 μM, respectively. The co-crystal structure analysis of these compounds in conjunction with JMJD3 (Fig. 4F−H) [175] reveals key interactions within the enzyme's catalytic domain. Specifically, compounds **19** and **20** established three hydrogen bonds with K1381, N1400, and R1246 in JMJD3's catalytic pocket, alongside a single chelating interaction with cobalt ion (Co^2+^). In contrast, compound **21**, while forfeiting its hydrogen bond with R1246, forms bidentate chelation with Co^2+^, mirroring the binding efficiency seen in potent inhibitors GSK-J1/J4. This distinct mode of interaction likely accounts for the enhanced inhibitory efficacy observed with compound **21** when compared to compounds 1**9** and **20** [175].

## 6 Conclusion and future perspectives

JMJD3 is a demethylase enzyme involved in regulating gene expression by removing H3K27me3 markers from chromatin. Its activation serves as an important host response to environmental and cellular stress, facilitating gene expression and being involved in physiological and pathological processes such as immune response, tissue regeneration, and adaptation. Therefore, understanding the regulation of JMJD3 expression and activity is vital for developing effective therapeutic interventions. Excessive expression of JMJD3 can lead to uncontrolled transcription and compromise nuclear integrity by reducing H3K27me3 levels. Importantly, JMJD3 is crucial in mediating the inflammatory reaction to cellular environmental shifts. While the demethylase function of JMJD3 warrants further exploration, especially its ambivalent influence on inflammation, certain tissues remain under-researched, and its synergy with other chromatin-altering complexes is vital for its *in vivo* role. Furthermore, investigations have confirmed roles for JMJD3 beyond demethylation, indicating that its function extends beyond simply removing methyl groups. All KDMs feature binding domains devoid of catalytic function, suggesting their interaction with chromatin or nucleosomes involves complex protein-protein or protein-nucleic acid engagements, including, methylation/demethylation processes. Consequently, focusing on the binding domains of KDMs could unveil new therapeutic avenues. Furthermore, JMJD3 induces PHF20 degradation in a non-dependent demethylase activity. Therefore, the non-dependent demethylase activity of JMJD3 should be investigated further.

JMJD3 plays a critical role in inflammation, making the development of selective and potent JMJD3 inhibitors an area of active interest. Currently, the most potent inhibitor available is compound **1**, which exhibits selectivity for KDM6 over the KDM5 sub-family. However, the lack of selective inhibitors has hindered a comprehensive understanding of JMJD3's biological functions in cellular processes. The cell-permeable prodrug GSK-J4, while less active, lacks selectivity *in vitro* and *in vivo* assays. Hence, there is an urgent need for more selective and potent JMJD3 inhibitors to elucidate JMJD3's role in physiological and disease processes and mitigate off-target effects. Several challenges arise in the development of JMJD3 inhibitors. For example, most identified inhibitors function as 2-OG competitors or metal-chelating agents. Moreover, the high similarity between the catalytic domains of JMJD3 and other demethylases, particularly within the JmjC family, complicates the development of selective inhibitors. Additionally, common challenges in inhibitor design and optimization, such as enhancing cell permeability and stability, need to be addressed. Furthermore, it is worth considering that inhibiting JMJD3 demethylase activity alone may not be sufficient to reduce the expression of target genes. Further investigations are required to fully understand the regulatory mechanisms involving JMJD3 and identify potential combinatorial approaches for effective gene expression modulation. Several solutions have been proposed to confront these challenges. Initially, specific non-catalytic domains (such as the GATAL domain) have been recognized as regulators of JMJD3 demethylase activity. By focusing on these less-conserved domains, it may be possible to specifically hinder the catalytic function of JMJD3, providing a means to selectively regulate its activity. In light of the enzymatic characteristics of JMJD3 demethylase, an alternative approach to targeting this enzyme involves pinpointing allosteric regulatory sites and creating allosteric inhibitors. Another aspect to consider in the development of JMJD3 inhibitors is improving their cell permeability and stability. By implementing medicinal chemistry principles, lead compounds can be optimized to enhance their properties and overall effectiveness as inhibitors. Additionally, exploring combination therapy holds promise for the clinical development of JMJD3 inhibitors, potentially expediting their use in a therapeutic setting. Furthermore, targeting the interaction between JMJD3 and its partner proteins could be a viable strategy to enhance the selectivity of JMJD3 inhibitors. Collectively, these strategies offer potential pathways for addressing the challenges associated with targeting JMJD3. Modulating PPI is a recognized strategy for enhancing the efficacy and selectivity of targeted therapies. Similar to other KDMs, JMJD3 interacts with various proteins to exert disease-promoting effects. Disruption of the PPI between JMJD3 and its ligand proteins is a promising avenue for improving the efficacy and selectivity of JMJD3 inhibitors.

In summary, JMJD3 serves as a pivotal factor in the modulation of inflammation by exerting its demethylase and non-demethylase activities. Targeting JMJD3 demethylase activity emerges as a potential therapeutic approach for addressing a spectrum of inflammatory conditions mediated by JMJD3. Nonetheless, there are instances where JMJD3 has been observed to suppress inflammation. Enhanced scrutiny into the pathological implications of JMJD3 in diverse inflammatory disorders and the customization of its inhibitors to suit these conditions are imperative for the effective implementation of JMJD3 inhibitors in clinical settings.

## CRediT author statement

**Xiang Li:** Investigation, Project administration, Visualization, Writing - Original draft preparation; **Ru-Yi Chen:** Formal analysis, Investigation, Project administration, Validation, Visualization, Writing - Original draft preparation; **Jin-Jin Shi**, **Chang-Yun Li**, and **Yan-Jun Liu:** Data curation, Formal analysis, Investigation, Validation, Visualization; **Chang Gao:** Data curation, Formal analysis, Funding acquisition, Project administration, Validation, Visualization; **Ming-Rong Gao:** Data curation, Formal analysis, Investigation, Methodology, Validation, Visualization; **Shun Zhang:** Formal analysis, Investigation, Validation, Visualization, Writing - Reviewing and Editing; **Jian-Fei Lu:** Formal analysis, Investigation, Project administration, Validation, Visualization, Writing - Original draft preparation; **Jia-Feng Cao:** Data curation, Formal analysis, Investigation, Project administration, Validation, Visualization, Writing - Reviewing and Editing; **Guan-Jun Yang:** Conceptualization, Funding acquisition, Investigation, Project administration, Resources, Software, Supervision, Writing - Reviewing and Editing; **Jiong Chen:** Conceptualization, Funding acquisition, Resources, Software, Supervision, Writing - Reviewing and Editing.

## Declaration of competing interest

The authors declare that there are no conflicts of interest.
